# Editorial: Unraveling plant-microbe interactions: from ecology to mechanisms, volume II

**DOI:** 10.3389/fpls.2023.1234372

**Published:** 2023-07-14

**Authors:** Essaid Ait-Barka, Brigitte Mauch-Mani

**Affiliations:** ^1^ Unité de Recherche Résistance Induite et BioProtection des Plantes- EA4707 - USC INRAe1488 UFR Sciences Exactes et Naturelles, Université de Reims Champagne-Ardenne, Reims, France; ^2^ Département de Biologie, Laboratoire de Biologie Moléculaire et Cellulaire, Université de Neuchâtel, Neuchâtel, Switzerland

**Keywords:** oomycete, nitric oxide, resistance genes, endophytes, *Verticillium* wilt

The co-evolutionary relationship between plants and microbes has led to complex mechanisms of offense and defense, primarily centering on the innate immune system of host plants countering the virulence determinants employed by pathogens (Webster, 2014). Both above ground and underground plant organs are frequently exposed to intimate contact with a wide range of microorganisms, including members of phyla as diverse as viruses, bacteria, oomycetes, fungi, and eukaryotic protozoans. The outcome of interactions between the plants and these microbial communities can be neutral, detrimental, or even beneficial for the plants.

The molecular events occurring between plants and both ‘friendly’ and ‘hostile’ microbes trigger a range of highly dynamic plant cellular responses within the plant. These responses play a crucial role in pathogen recognition and the induction of appropriate signaling pathways for effective defense (Peyraud et al., 2017).

The Research Topic “*Unraveling Plant-Microbe Interactions: From Ecology to Mechanisms, Volume II*” encompasses various aspects of plant-microbe interactions and explores several intriguing questions. These include: why do certain microbes target specific plants while ignoring others? Is it due to the lack of necessary tools for infecting certain plants, or do some plants possess superior defense mechanisms to counteract such attacks? Is there a lack of attraction between the microbe and the plant? How do plants defend themselves in response to pathogen attacks? What mechanisms do they employ to recognize and counteract pathogens? How do plants interact with beneficial microorganisms? What are the molecular events and signaling pathways involved in establishing symbiotic relationships between plants and helpful microbes? Finally, how do plant pathogens respond to antagonistic microorganisms, and how does this response impact the effectiveness of biocontrol strategies?

Oomycetes, including *Phytophthora* (Erwin and Ribeiro, 1996) and Plasmopara (Koledenkova et al., 2022), pose significant and grave risks to plants, as emphasized in the study by Derevnina et al. (2016). These pathogens have the potential to inflict substantial losses on various plant species. This context sets the stage for the following three articles, which delve into novel perspectives and provide valuable insights into the intricate ways plants respond to oomycete attacks.

To better understand the events taking place during the infection of potato plants with the late blight pathogen *Phytophthora infestans*, Li et al. took advantage of the dual RNA-seq technique to study the genome-wide expression profiles in a late blight–resistant and susceptible cultivar. The study identified distinct infection profiles for effector, NBS-LRR, and kinase-encoding genes in the compatible and the incompatible interaction.

Drozda et al. investigated the influence of nitric oxide (NO) on the expression of resistance (R) genes regulated by the RNA-directed DNA methylation pathway (RdDM). Specifically, the authors studied the role of NO-dependent redox targets involved in the tuning of the *R gene* expression by complementary miRNA during the potato-avr *P. infestans* interaction. The researchers proposed that biphasic waves of NO released in response to *P. infestans* play a crucial role in the successful establishment of resistance to late blight in potato through the RdDM pathway controlling *R gene* expression.

In their study, Philosoph et al. explored the response of cucumber plants to co-infection with two distinct pathogens: *Pythium spinosum*, a necrotrophic pathogen, and *Cucumber green mottle mosaic virus* (CGMMV), which belongs to a different kingdom and has a different lifestyle. An intriguing finding from their research is the rapid downregulation of host defense gene activity associated with defense against necrotrophic pathogens following the establishment of the virus in plants previously infected with *Pythium*. The study by Philisoph and colleagues provides valuable insights into the complex interactions and regulatory processes involved when plants face the challenge of co-infection by pathogens from different kingdoms and with distinct lifestyles.

In addition to their role in defense against pathogens, plant defense mechanisms also play a role in the case of endophytes. *Epichloë*, a fungal endophyte, is known to colonize cool-season grasses (*Poaceae*) (Selosse and Schardl, 2007). Zhang et al. studied seed transmission of *Epichloë* in perennial ryegrass, focusing on the transcriptomes of reproductive tissues from host genotype with high-transmission and low-transmission rates. Their research revealed that the efficiency of seed-transmission of the endophyte is mainly influenced by plant defense responses lowering the colonization of host tissues by *Epichloë*. The findings highlight the significance of plant defense mechanisms in modulating the establishment and transmission of endophytes such as *Epichloë* in cool-season grasses.

The incidence of *Verticillium* wilt, a devastating disease affecting olive trees, poses significant challenges in olive-growing regions worldwide, and its control is notoriously difficult (Tsror, 2011). In an effort to better understand the mechanisms underlying olive tree tolerance to this pathogen, Cardoni et al. employed a comprehensive approach. Using different olive varieties with varying degrees of susceptibility/tolerance to *Verticillium* wilt, the authors investigated multiple aspects, including root functional traits, expression of defense-related genes, root lignin content, and membrane permeability. Through their research, the authors successfully identified specific factors that influence the performance of olive trees when confronted with *Verticillium* invasion.

Finally, in their review article on the potential role of the microbiome in managing Hualongbin disease in citrus caused by *Candidatus Liberibacter asiaticus* (Das et al., 2021), Srivastava et al. present the knowledge, prospective methodology, and open questions remaining in this area of research.

Overall, the articles included in this Research Topic further validate the extensive range of defense mechanisms that plants possess to safeguard themselves against various threats.

## Author contributions

All authors listed have made a substantial, direct, and intellectual contribution to the work and approved it for publication.
